# Regional variances depict a unique glial-specific inflammatory response following closed-head injury

**DOI:** 10.3389/fncel.2023.1076851

**Published:** 2023-02-15

**Authors:** Michelle R. White, Pamela J. VandeVord

**Affiliations:** ^1^Department of Biomedical Engineering and Mechanics, Virginia Tech, Blacksburg, VA, United States; ^2^Salem VA Medical Center, Salem, VA, United States

**Keywords:** astrocytes, aquaporin (AQP) 4, microglia, concussion, closed-head controlled cortical impact, closed-head injury

## Abstract

Mild traumatic brain injuries (mTBI) constitute a significant health concern with clinical symptoms ranging from headaches to cognitive deficits. Despite the myriad of symptoms commonly reported following this injury, there is still a lack of knowledge on the various pathophysiological changes that occur. Preclinical studies are at the forefront of discovery delineating the changes that occur within this heterogeneous injury, with the emergence of translational models such as closed-head impact models allowing for further exploration of this injury mechanism. In the current study, male rats were subjected to a closed-head controlled cortical impact (cCCI), producing a concussion (mTBI). The pathological effects of this injury were then evaluated using immunoflourescence seven days following. The results exhibited a unique glial-specific inflammatory response, with both the ipsilateral and contralateral sides of the cortex and hippocampus showing pathological changes following impact. Overall these findings are consistent with glial changes reported following concussions and may contribute to subsequent symptoms.

## Introduction

Traumatic brain injury (TBI) is prevalent among civilian and military populations, with high morbidity, mortality, and economic costs (Albert-Weissenberger and Sirén, 2010; Wilk et al., 2012; Faul and Coronado, 2015; Capizzi et al., 2020). Impact TBIs occur when an object strikes the head or when the head strikes a surface (McAllister, 2011; Meaney and Smith, 2011; Lizzo and Waseem, 2022). These forces can result in tissue deformation from compressive and shear forces within the brain, leading to increased intracranial pressure (ICP) (Bayly et al., 2005; Post, 2014; Stemper et al., 2015). TBIs are heterogeneous and have been delineated into three severities: mild, moderate, and severe. Mild TBIs (mTBIs) are commonly reported and are referred to as concussions (Kamins and Giza, 2016; Fetta et al., 2023). Concussive injury can be sustained from many events, such as vehicular accidents, military conflicts, and contact sports, such as football or soccer. Clinical symptoms of concussions include headaches, balance problems, dizziness, memory deficits, insomnia, anxiety, and/or depression (Wilk et al., 2012; Silverberg et al., 2013; Tator, 2013; Bolouri and Zetterberg, 2015; Stone et al., 2015; Ferry and DeCastro, 2022; Fish et al., 2022; Kraemer et al., 2022; Terpstra et al., 2022). While concussions (mTBIs) are the most common type of TBI, the exact mechanisms of concussions and what leads to the myriad of symptoms associated with this injury are still not understood.

Using preclinical models for TBI research is crucial in understanding the injury mechanisms, with closed-head impact injury systems coming to the forefront as clinically relevant. Since this is a relatively new model, previous literature using this model has yet to define the injury, with its characterization focusing on neuronal dysfunction in response to injury (Shitaka et al., 2011; Prins et al., 2013; Jamnia et al., 2017; McNamara et al., 2020). Glial cells are vital players in the injury response as their dysfunction can trigger blood-brain barrier (BBB) disruption, oxidative stress, and inflammatory responses (Abbott et al., 2006; Acosta et al., 2013; Khatri et al., 2018). Glial-driven pathology can result in cell death, breakdown of post-synaptic structures and axonal damage, neuroplasticity, and phagocytosis of cell debris. Astrocytes and microglia are glial cells that play critical roles in these consequences. As the most abundant cell type in the central nervous system (CNS), astrocytes become hypertrophic in response to injury and have been shown to express increased levels of aquaporin 4 (AQP4), a protein in their endfeet that interacts with blood vessels, playing a crucial role in BBB integrity (Yao et al., 2015; Ikeshima-Kataoka, 2016; Mira et al., 2021). Furthermore, astrocytes become reactive, existing in both pro- (A1) and anti-inflammatory (A2) activation states which can lead to upregulation of molecules such as tumor necrosis factor (TNF), interleukin 1α (IL-1α), and complement component 1q (C1q), controlling the balance between neuroprotective and neurotoxic properties (Giovannoni and Quintana, 2020). A hallmark of astrocyte reactivity is the increased expression of glial fibrillary acidic protein (GFAP) and vimentin. Novel pathways that signal astrocyte reactivity can lead to the recruitment of peripheral immune cells, allowing astrocytes to sense and respond to pro-inflammatory cytokines secreted by peripheral immune cells or the CNS-resident immune cells, microglia (Linnerbauer et al., 2020).

Microglia are essential in the inflammatory response as they have diverse activation states. Traditionally, microglia have been known to polarize into “m1” (pro-inflammatory) and “m2” (anti-inflammatory) states, but recent studies have shown that changes in their morphology are more indicative of their specific activation state (Cao et al., 2012; Cherry et al., 2014; Young and Morrison, 2018). Studies have linked the activation of the Nucleotide-Binding Domain (NOD)-Like Receptor Protein 3 (NLRP3) inflammasome within glial cells, especially microglia, to an increase in pro-inflammatory cytokine production which can trigger activation of astrocytes (Feng et al., 2015; Grebe et al., 2018; He et al., 2019; Américo-Da-Silva et al., 2021). While this response is exacerbated early following focal brain injuries, there are few closed-head studies that have explored the influences of NLRP3, making it imperative to explore its response in a clinically relevant model. A study that explored microglia activation in post-mortem brains of TBI patients indicated that shear forces directly influence microglia morphology (Lier et al., 2020). With the limited studies on the robust analysis of microglia morphology following injury (Lafrenaye et al., 2015; Ryu et al., 2021), continuing work that defines the injury through microglia-mediated responses may lead to the generation of data that is useful in developing diagnostic tools and therapeutic interventions. The emerging role of the interplay among astrocytes and microglia has been observed, and their responses to injury are varied, finely regulated, and region-specific (Lana et al., 2021). Moreover, due to impact loading, the brain’s rotational and linear acceleration forces produce a disordered metabolic cascade or biochemical injury that may influence diverse microglia and astrocyte reactivity states that are unique to impact injury (Tator, 2013). Thus, advancing studies that explore the underlying mechanisms that control neuropathological changes in TBI are essential.

For this study, we chose to use the closed-head controlled cortical impact (cCCI) model because of its advantages over other preclinical TBI models, such as the open-head CCI and fluid percussion injury (FPI), which require a craniotomy, lacking translational aspects; and the weight drop model, whose limitations involve difficulties in reproducibility. This study aimed to examine the cellular and molecular changes following a closed-head injury model (CHI) of concussion with the impact being on the skin’s surface (closed-skull), not producing skull fracture or other obvious signs of injury (invisible nature), adapted from studies utilizing closed-head CCI devices (Prins et al., 2013; Jamnia et al., 2017). We hypothesized that this model would produce mild TBI with clinically relevant outcomes and that glial activation pathology would persist sub-acutely, yet the level of response would depend on the brain location, leading to region-specific increases of neuroinflammation.

## Materials and methods

### Animal procedures and closed-head controlled cortical impact (cCCI)

The Virginia Tech Institutional Animal Care and Use Committee (IACUC) approved the experimental protocols described herein. Before experimentation, 12 week old male Sprague Dawley rats (Envigo, Dublin, VA, USA) weighing approximately 300–330 g were acclimated for several days (12 h light/dark cycle) with food and water provided *ab libitum.*

**FIGURE 1:**
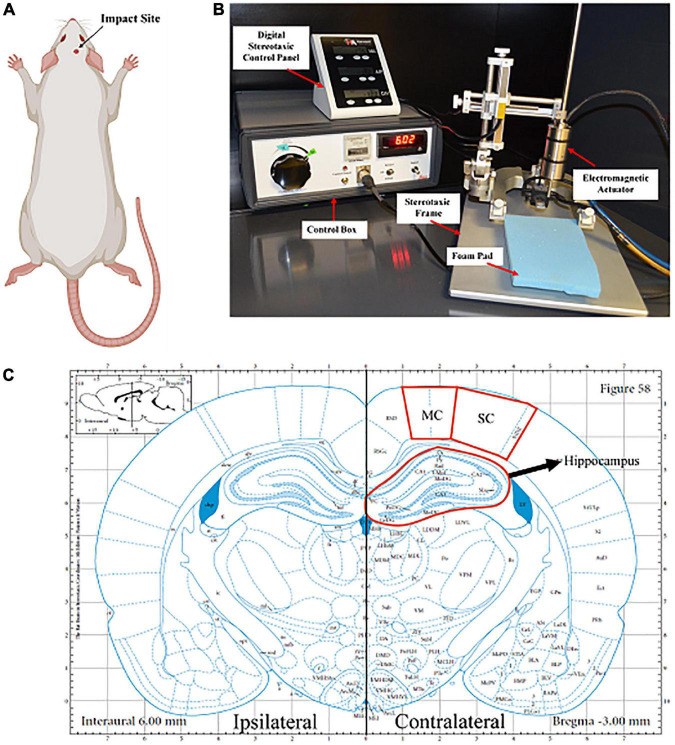
Closed head controlled cortical impact. **(A)** The impact area was on the right side of the rat’s head, specifically on the right parietal bone, above the somatosensory cortex. Created with BioRender.com. **(B)** The impactor was mounted onto the stereotaxic frame, which aligns with the region of interest. A foam pad was placed under the animal’s head, as the head was not attached to the ear bars. The impactor was connected to the control box where the dwell time and velocity were set. **(C)** Representative image of the coronal section of the brain that is –3.00 mm posterior from Bregma. The somatosensory cortex (SC), motor cortex (MC), and hippocampus on both the ipsilateral and contralateral sides of the brain are located within this coronal section.

An impactor device induced a mild/moderate closed head injury in rats (*n* = 5). Under 5% isoflurane, animals were placed in the stereotaxic frame where the head was shaven, teeth were placed in the teeth bar, and the nose was placed in the stereotaxic nose cone to maintain anesthesia. A foam pad (∼2 cm thick) was situated within the stereotaxic frame, where the animal’s head was laid against without ear bars to ensure that the head could freely move in the direction of the injury. An electromagnetic piston-driven actuator was mounted onto the stereotaxic crossbar to allow for defined localization of the impact center (Figures 1A, B). A skull template was used to mark the center of impact (right somatosensory cortex (SC), ∼3–5 mm posterior to bregma), and the injury tip was firmly zeroed against the skin. The impactor (tip diameter: 5 mm) created a force at a velocity of 6 m/s at a depth of 8 mm and a dwell time of 300 ms inducing injury (Impact One, Leica Biosystems, Buffalo Grove, IL). Immediately following impact, animals were then placed on a heating pad at 37°C, monitored, and the time it took for them to right was recorded (righting reflex). Sham animals underwent all the same procedures except the impact (*n* = 6).

### Tissue processing

Seven days following injury, animals were euthanized by transcardial perfusion of 0.9% saline and 4% paraformaldehyde. Following perfusion, brains were collected and stored in a 4% paraformaldehyde fixative solution for an additional 24 h. Following fixation, brains were cryoprotected in 30% sucrose solution for tissue sectioning preparation. Once whole brains were submerged entirely in sucrose solution (∼48 h), tissues were embedded in Tissue-Tek optimal cutting temperature (OCT) embedding medium (Sakura Finetek US, Inc., Torrance CA) and frozen at −80^°^C until cryostat sectioning. Brains ready for cryosectioning were sectioned coronally with a cryostat at a thickness of 30 μm. Sections were collected serially in 12-well plates and stored in phosphate-buffered saline (PBS) with 0.05% sodium azide. Sections containing the motor and somatosensory cortices (MC and SC, respectively) and the hippocampus (∼−3.00 mm posterior from bregma) were randomly selected and used for histological analyses (Figure 1C).

### Immunofluorescence

Sections were stained for the following antibodies: Aquaporin 4 (AQP4), Glial Fibrillary Acidic Protein (GFAP), ionized calcium-binding adaptor molecule 1 (IBA1), or NLR family pyrin domain containing 3 (NLRP3) (Table 1). More specifically, free-floating sections were washed three times for 5 min each in 1X PBS and then permeabilized in PBS with 0.3% Triton X (PBX) for 30 min at room temperature. Samples were blocked in either 2% bovine serum albumin (AQP4, GFAP, or IBA-1) or 5% Normal Donkey Serum (NLRP3) in PBS for 1 h at room temperature. Once samples were permeabilized and blocked, they were incubated for 16–18 h at 4°C with a primary antibody. The following day, sections were washed three times for 5 min in PBX and incubated for 1.5 h at room temperature with secondary antibodies Alexa Flour 488 Goat anti-mouse IgG antibody (Invitrogen, Carlsbad, California) (GFAP) or Alexa Flour 546 Goat anti-rabbit IgG antibody (Invitrogen, Carlsbad, California) (AQP4, IBA-1, and NLRP3). After three more 5-min PBX washes, samples were mounted and coverslipped with Slow Fade Reagent with DAPI (Invitrogen, Carlsbad, CA). Sections were then imaged using a Zeiss fluorescence microscope at 20X magnification by an investigator blinded to animal groups.

**TABLE 1:** Primary antibodies used for tissue histology.

Antibody	Catalog number	Target	Vendor
AQP4 (1:250)	sc32739	Water channels	Invitrogen (Carlsbad, CA)
GFAP (1:500)	13–300	Astrocytes	Invitrogen (Carlsbad, CA)
IBA-1 (1:300)	CP290B	Microglia	Biocare (Concord, CA)
NLRP3 (1:200)	PA5-79740	Inflammasomes	Invitrogen (Carlsbad, CA)

### Image acquisition and analyses

Images were acquired at each ipsilateral and contralateral region and then processed with background subtraction and fixed thresholding to generate masks of IBA-1 + microglia, GFAP + astrocytes, or cells containing AQP4 or NLRP3 (Figure 1C). Using FIJI/Image J software, a comprehensive analysis of neuropathology was provided by quantifying four specific parameters using ImageJ software (NIH, Bethesda, MD). These parameters include area fraction, count per area, fluorescence intensity, and area per cell. Area fraction quantifies the percentage of positive signal within the region of interest. Count per area represents the total number of positive cells divided by the area. Fluorescence intensity measures changes in the positive signal of the cells using the gray pixel intensity. Mean area per cell provides detail to the average cell size normalized to the area, giving the average area of the cell. Count per area and area per cell was completed using the “analyze particles” function with the pixel area size threshold of 25 to exclude small pixel noise and extract objects of interest. Brain region values were averaged from four images for each animal per stain.

### Quantifying microglia morphology

The skeleton analysis method was used to further determine microglia activation by quantifying microglia morphology (Young and Morrison, 2018). Images were converted to binary and skeletonized using ImageJ software. The AnalyzeSkeleton plugin was then applied to all skeletonized images to collect data on the number of endpoints and processes length per frame. The data from each frame was then divided by the number of microglia somas in the corresponding image to give branch length per cell and branch points per cell.

### Statistical analyses

All statistical analyses were performed in GraphPad Prism Version 9 (GraphPad Software, La Jolla, CA). The Kolmogorov–Smirnov test, in association with the Shapiro–Wilk Test, was performed to test for normal distribution and equal variance of the data. Outliers were identified by calculating the studentized residuals, excluding data points above −2 or 2. If the data passed the two assumptions, a one-way ANOVA and *post-hoc* tests were performed where appropriate for sham, cCCI contralateral, and cCCI ipsilateral groups. Data that did not pass normality or equal variance assumptions were assessed using a one-way Kruskal–Wallis test and/or pairwise Mann–Whitney *U*-test. All data is represented as the mean ± standard error of the mean, or SEM.

## Results

### Animal recovery and righting reflex

**FIGURE 2:**
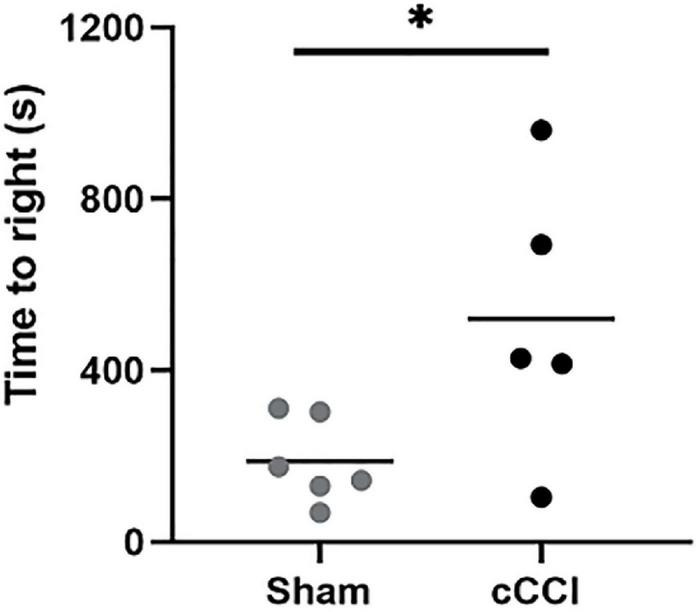
Injury outcomes were quantified by measuring time to right. Righting reflex was measured immediately following impact. Compared to shams, cCCI animals showed significant delays in righting time. *p < 0.05. Data represented as Mean ± SEM.

Immediately following impact, animals were placed on their backs on a heating pad, where time to right (righting reflex) was measured. Animals subjected to cCCI showed significant delays in their time to right compared to the sham group (*p* = 0.03). Yet, there were no other apparent signs of injury (Figure 2). Over 7 days, although decreased, there was no significant difference in weights observed in the cCCI group compared to the sham group. The average weight of the cCCI group at day 7, was 296 g ± 22.3, while the average weight of the sham group was 337.4 g ± 7.16.

### Astrocyte reactivity was observed within the SC, with limited changes in the MC of animals seven days following impact

**FIGURE 3:**
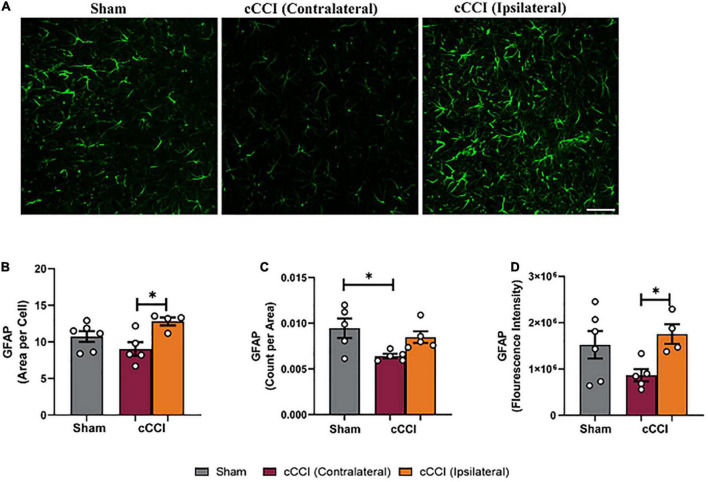
Changes in astrocytes were notable within the SC 7 days following impact. **(A)** Representative images of GFAP in the SC region of the brain. Magnification is at 20× and scale bar = 100 μm. **(B)** Decreases in area per cell were observed in the contralateral SC compared to the ipsilateral side in the cCCI animals. **(C)** Significant decreases in the number of GFAP + astrocytes (count per area) were observed in the contralateral SC of the cCCI group compared to the sham animals. **(D)** Decreased levels of GFAP (fluorescence intensity) were found in the contralateral SC compared to the ipsilateral SC in the injured group. *p < 0.05. Data represented as Mean ± SEM.

A one-way ANOVA indicated a significant increase in the size of GFAP + astrocytes (area per cell) in the ipsilateral side of the somatosensory cortex in cCCI animals compared to the contralateral side (*p* = 0.02). No significant differences were observed when comparing the ipsilateral or contralateral side of the injured group to shams. The number of GFAP + astrocytes (count per area) was significantly decreased in the contralateral side of the somatosensory cortex in injured animals compared to shams (*p* = 0.03). When observing fluorescence intensity, *post-hoc* tests indicated a significant increase in the ipsilateral side of the SC compared to the contralateral side within the cCCI group (*p* = 0.03) (Figures 3A–D). No significant differences were found when observing increases or decreases in area fraction of GFAP within the SC. Additionally, no significant differences were found for any parameter within the MC region.

### Astrocyte pathology was notable in the hippocampus of cCCI animals at seven days

**FIGURE 4:**
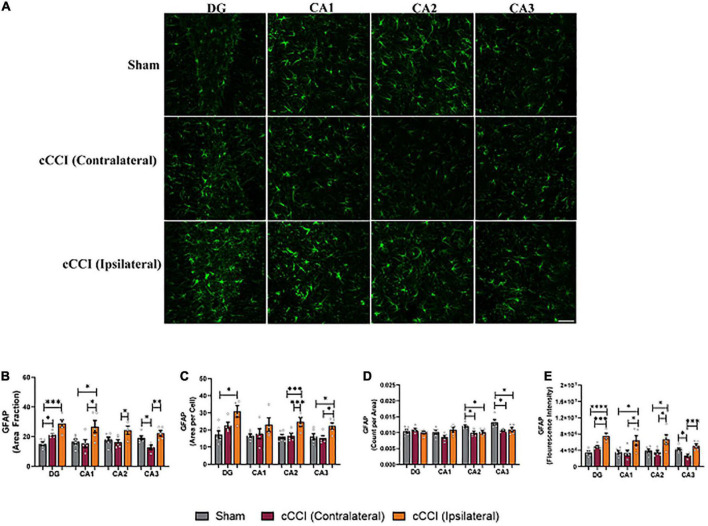
Closed-head controlled cortical impact induced subacute astrocyte reactivity in the hippocampus. **(A)** Representative images of GFAP + astrocytes in the hippocampus. Magnification at 20×, scale bar = 100 μm. **(B)** Significant increases in area fraction were found in both the contralateral and ipsilateral sides of the DG of the cCCI animals compared to the DG of sham group. Area fraction was also increased within the ipsilateral side of the CA1, CA2, and CA3 sub-regions compared to the contralateral side of the injured group. Additionally, within the CA1 sub-region, there was a significant increase in area fraction in the ipsilateral side in the injured group compared to shams. The contralateral CA3 in the cCCI animals showed notable decreases in area fraction compared to their sham counterparts. **(C)** Significant increases in area per cell were notable in the ipsilateral DG of the cCCI group compared to shams. Significant increases in area per cell were also observed in the CA2 and CA3 sub regions of the cCCI animals compared to both the contralateral side in the cCCI group and the CA2 and CA3 sub-regions in the sham animals. **(D)** Decreases in count per area were notable in the CA2 and CA3 sub-regions in the sham animals compared to both the ipsilateral and contralateral sides in the cCCI group. **(E)** Elevated levels of GFAP (fluorescence intensity) were observed in the ipsilateral side of the hippocampus compared to the contralateral side of the impacted animals. Elevated levels of GFAP were also observed when comparing the ipsilateral side of all sub-regions of the hippocampus in the cCCI animals compared to shams. *p < 0.05, ^**^p < 0.01, ^***^p < 0.001, and ^****^p < 0.0001. Data represented as Mean ± SEM.

Changes in GFAP expression were evident within the hippocampus, with notable changes within each sub-region (Figure 4A). Significant increases in the percentage of positive signal of GFAP (area fraction) were observed in the contralateral side of the DG in the injured group compared to the DG in the sham animals (*p* = 0.02). The ipsilateral DG within the cCCI group was also significantly higher compared to shams (*p* = 0.0003). *Post-hoc* tests also indicated similar trends in the CA1 as well, where area fraction was significantly higher on the ipsilateral side compared to the contralateral side of the cCCI group (*p* = 0.01), as well as when comparing the ipsilateral CA1 of the cCCI group to the sham group (*p* = 0.02). The CA2 sub-region indicated that the area fraction of GFAP was significantly higher in the ipsilateral side of the brain in the cCCI animals compared to the contralateral side (*p* = 0.04). Significant decreases in area fraction of GFAP were also observed in the contralateral side of the CA3 compared to the ipsilateral side in cCCI animals (*p* = 0.004). This decreases was also found when comparing the contralateral CA3 in the cCCI group to the CA3 of the shams (*p* = 0.04) (Figure 4B).

Increases in the cell size of GFAP + astrocytes (mean area per cell) were significantly increased in the ipsilateral side of the DG compared to the DG of shams (*p* = 0.03). The ipsilateral side of the CA2 sub-region of the hippocampus showed significant increases in cell size compared to both the contralateral side of the CA2 in cCCI animals (*p* = 0.007) and the sham group (*p* = 0.004). Significant changes were observed in the CA3 sub-region, with area per cell significantly increased on the ipsilateral side compared to the contralateral side (*p* = 0.02). The ipsilateral CA3 of the injured group also significantly increased compared to shams (*p* = 0.04). The amount of GFAP + astrocytes was significantly decreased when comparing the ipsilateral CA2 in the injured animals to shams (*p* = 0.01), as well as contralateral CA2 in the cCCI group to the CA2 within the sham animals (*p* = 0.02). A similar significant decrease was observed in the CA3 sub-region with the contralateral side of the CA3 in the injured group significantly decreased compared to shams (*p* = 0.01), as well as the ipsilateral CA3 in the injured group decreased compared to shams (*p* = 0.02) (Figures 4C, D).

A one-way ANOVA showed significant increases in GFAP expression (fluorescence intensity) with *post-hoc* tests indicating that the ipsilateral side of the DG was increased compared to the contralateral side of the brain in the cCCI group (*p* = 0.004). The ipsilateral DG of the cCCI group was also increased compared to the DG of the sham animals (*p* < 0.0001). Elevated levels of GFAP were also observed in the CA1, with the ipsilateral side of the injured brain significantly higher when compared to the contralateral side of the brain in cCCI animals (*p* = 0.01). The ipsilateral side of the CA1 in the injured group was significantly increased when compared to shams (*p* = 0.01). Similar outcomes were observed in the CA2 sub-region with fluorescence intensity significantly higher on the ipsilateral side compared to the contralateral side of the brain in cCCI animals (*p* = 0.02), and the ipsilateral CA2 of the injured group was also significantly elevated compared to shams (*p* = 0.03). Within the CA3 sub-region, fluorescence intensity was significantly decreased on the contralateral side of the brain compared to the ipsilateral side of the cCCI group (*p* = 0.004). The contralateral CA3 within the cCCI animals showed lower fluorescence intensity compared to shams (*p* = 0.04) (Figure 4E).

### Elevated levels of AQP4 were observed in the MC and hippocampus, but not the SC following injury

**FIGURE 5:**
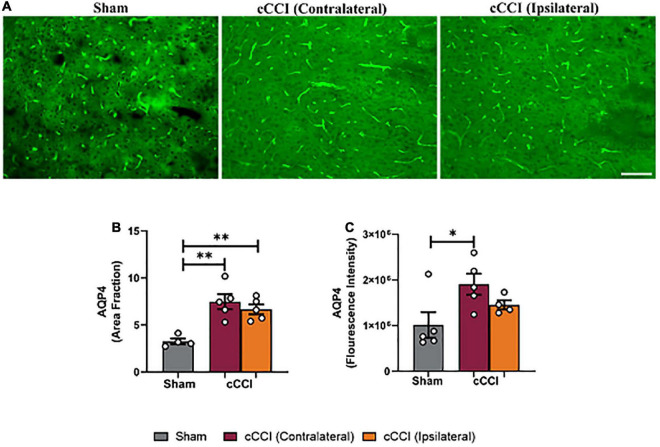
Closed-head controlled cortical impact increased levels of AQP4 in the MC at 7 days. **(A)** Representative images show increased levels of AQP4 in the injured group. Magnification = 20×. Scale bar = 100 μm. **(B)** Significant increases in area fraction of AQP4 were found within the ipsilateral and contralateral sides of the MC of the cCCI group compared to the MC of their sham counterparts. **(C)** A significant increase in fluorescence intensity was observed in the contralateral side of the MC within the injured group compared to the MC of the shams. *p < 0.05, ^**^p < 0.01. Data represented as Mean ± SEM.

Increases in AQP4 expression were found to be significant following cCCI (Figure 5A). A one-way ANOVA indicated a significant change in area fraction of AQP4 in the MC. More specifically, *post-hoc* tests indicated that the area fraction of AQP4 was significantly higher in the contralateral of the MC in the injured group compared to the MC in shams (*p* = 0.007), as well as the ipsilateral side of the MC in the injured group compared to shams (*p* = 0.001). Similar trends were observed when analyzing fluorescence intensity within the MC, with the contralateral side of the MC in the cCCI group showing significant increases in AQP4 expression compared to the sham group (*p* = 0.04) (Figures 5B, C). No significant changes were found for either area fraction or fluorescence intensity when analyzing the SC region.

**FIGURE 6:**
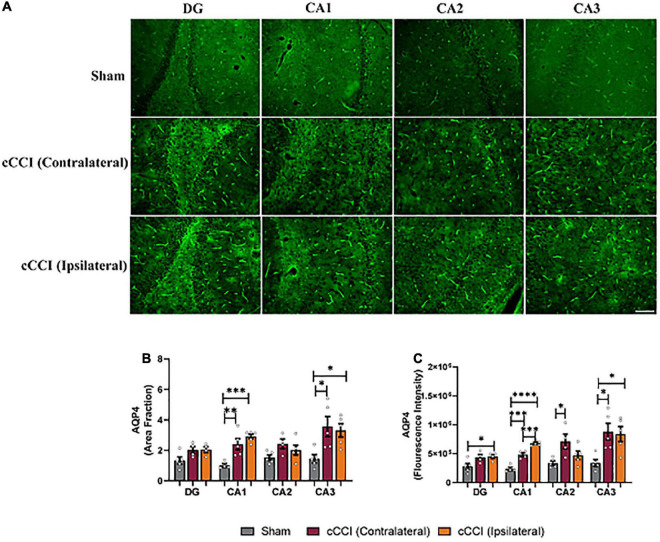
Upregulation of AQP4 was notable in the hippocampus 7 days following impact. **(A)** Representative images depict changes in AQP4 levels within the hippocampus of the injured group. Magnification = 20×. Scale bar = 100 μm. **(B)** Increases in area fraction were found in both the ipsilateral and contralateral sides of the CA1 and CA3 sub-regions of the hippocampus in the injured group compared to the same regions within shams. **(C)** Fluorescence intensity depicted significant increases in the ipsilateral DG in the cCCI animals compared to the DG of shams. Within the CA1 sub-region, the ipsilateral side was increased compared to the contralateral side in the cCCI group and the CA1 of the sham group, whereas the contralateral CA1 in the injured group was significantly increased compared to the CA1 in the sham group. Increases in fluorescence intensity was also noted within the contralateral side of the CA2 in the cCCI group compared to the CA2 in the sham group. Within the CA3 sub-region, the contralateral and ipsilateral sides of the cCCI animals were significantly elevated compared to the CA3 of the sham group. *p < 0.05, ^**^p < 0.01, ^***^p < 0.001, and ^****^p < 0.0001. Data represented as Mean ± SEM.

Within the CA1 sub-region of the hippocampus, significant increases in AQP4 were observed in cCCI animals compared to the sham group. *Post-hoc* tests showed that area fraction in the contralateral CA1 of the injured group was significantly elevated when compared to the CA1 within their sham counterparts (*p* = 0.007). A significant increase in area fraction was also present in the ipsilateral side of the CA1 in the injured group, compared to the CA1 of the shams (*p* = 0.0007). Within the CA3 sub-region, increases in area fraction of AQP4 was found in the contralateral side of the brain in the cCCI animals compared to shams (*p* = 0.02), and was significantly higher in the ipsilateral CA3 of the cCCI group compared to shams (*p* = 0.04). When analyzing fluorescence intensity, the ipsilateral side of the DG sub-region of cCCI animals was significantly increased compared to the DG in the sham group (*p* = 0.04). In the CA1 sub-region, elevated levels of AQP4 were observed within cCCI animals, with the ipsilateral side of the sub-region significantly higher than the contralateral side of the brain of the cCCI group (*p* = 0.0008). These levels were significantly decreased in the CA1 sub-region of sham animals when compared to both the contralateral and ipsilateral sides of the CA1 of the injured group (*p* = 0.0002, *p* < 0.0001, respectively). The contralateral side of the CA2 sub-region within the injured group indicated significant increases in fluorescence intensity of AQP4 compared to sham animals (*p* = 0.02), with no significant differences observed between the contralateral and ipsilateral sides of the brain in cCCI animals. Within the CA3, fluorescence intensity of AQP4 in the contralateral side of this sub-region in the cCCI group was significantly increased compared to shams (*p* = 0.02), this increase was also observed as fluorescence intensity in the ipsilateral CA3 of the injured group was also significantly higher than the CA3 within the sham animals (*p* = 0.02) (Figures 6A–C).

### Microglia activation was present in the MC and the hippocampus, with no changes present in the SC seven days following impact

**FIGURE 7:**
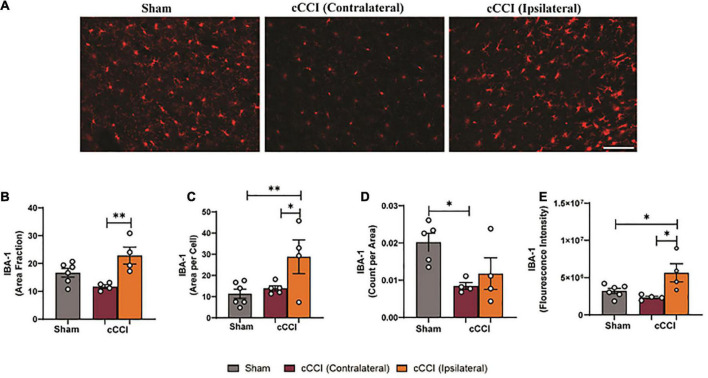
Altered levels of IBA-1 were found in the MC 7 days following cCCI. **(A)** Representative images show IBA1 + microglia within the MC of both shams and cCCI-injured animals. Magnification = 20×. Scale bar = 100 μm. **(B)** Increases in area fraction were notable in the ipsilateral side of the MC compared to the contralateral side of the injured animals. **(C)** Increases in the size of microglia (area per cell) were found to be significant in both the ipsilateral and contralateral sides of the MC in the injured animals compared to the MC of the shams. **(D)** Significant decreases in count per area was observed in the contralateral MC of the injured group compared to the MC of shams. **(E)** Significant increases in fluorescence intensity of IBA-1 were observed in the ipsilateral MC compared to the contralateral side of the injured group. The ipsilateral side of the MC in the injured group also exhibited increased fluorescence intensity compared to the MC in the shams. *p < 0.05 and ^**^p < 0.01. Data represented as Mean ± SEM.

Changes in levels of IBA-1 were observed in the MC following cCCI, indicating that microglia activation may be taking place (Figure 7A). A one-way ANOVA indicated that the area fraction of IBA-1 was significantly increased in the MC 7 days following injury (*p* < 0.05). *Post-hoc* tests showed increases in the ipsilateral side of the MC compared to the contralateral side in cCCI animals (*p* = 0.007). Increases in cell body size of IBA-1 + microglia (area per cell) were found, with significant increases in mean area per cell in the ipsilateral side of the MC compared to the contralateral side in cCCI animals (*p* = 0.02), and the ipsilateral side of the cCCI group compared to the MC in the sham group (*p* = 0.009). Significant decreases in the number of IBA-1 + microglia (count per area) were observed in the MC, with a significant decrease in count per area in the contralateral side of the MC of cCCI animals compared to the MC of the sham group (*p* = 0.04). No significant changes in count per area were found between the contralateral and ipsilateral sides of the MC in cCCI animals or between the ipsilateral side of the MC in the cCCI group and the MC of the shams. When observing fluorescence intensity, significant increases were found when comparing the ipsilateral side of the MC to the contralateral side in cCCI animals (*p* = 0.01). A significant increase was also notable when comparing the ipsilateral side of the MC of the cCCI group to shams (*p* = 0.02) (Figures 7B–E). No significant changes were observed for any parameter between the cCCI and sham animals when analyzing microglial activation in the SC region.

**FIGURE 8:**
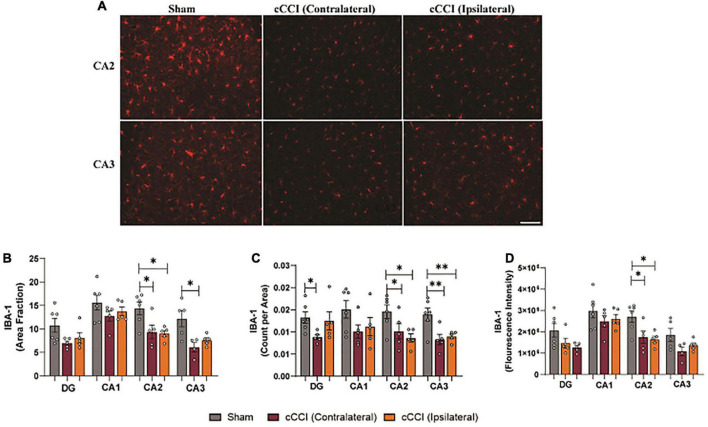
Microglia pathology was notable in the hippocampus following impact. **(A)** Representative images show changes in IBA-1 levels within the CA2 and CA3 sub-regions of the hippocampus in the injured group. Magnification is at 20× and scale bar is at 100 μm. **(B)** Decreases in area fraction were significant when comparing both the ipsilateral and contralateral sides of the CA2 sub-region in the injured animals compared to the CA2 within the shams. In the CA3 sub-region, there was a notable decrease in area fraction in the contralateral side in the cCCI group compared to shams. **(C)** Significant decreases in count per area were notable in the contralateral DG of the cCCI group compared to shams, while these significant decreases were found within both the contralateral and ipsilateral sides of the CA2 and CA3 of the injured animals compared to their sham counterparts. **(D)** In the CA2 sub-region, significant decreases in fluorescence intensity were observed in the contralateral and ipsilateral sides in the cCCI animals compared to the CA2 in the sham group. *p < 0.05 and ^**^p < 0.01. Data represented as Mean ± SEM.

Within the hippocampus, decreases in microglia and IBA-1 expression were notable (Figure 8A). A one-way ANOVA showed significant changes in area fraction of IBA-1, with *post-hoc* tests indicating that the contralateral side of the CA2 sub-region in the cCCI group was significantly decreased compared to shams (*p* = 0.02). This was also noted in the ipsilateral CA2 of the injured group as area fraction was significantly decreased compared to shams (*p* = 0.02). Within the CA3 sub-region, area fraction was significantly decreased on the contralateral side in the injured group compared to shams (*p* = 0.01). Decreases in IBA-1 + microglia were observed in the contralateral DG of the cCCI group compared to the DG of shams (*p* = 0.04). In the CA2 sub-region, count per area was significantly decreased in the contralateral side of the injured group compared to the CA2 within the shams (*p* = 0.04). This significant decrease was also found when comparing the ipsilateral side of the CA2 in the cCCI group to shams (*p* = 0.01). Count per area was also significantly different between groups in the CA3 sub-region, with both the contralateral and ipsilateral CA3 in the injured group significantly decreased compared to shams (*p* = 0.004; *p* = 0.009, respectively). Decreased IBA-1 expression (fluorescence intensity) was observed when comparing both the contralateral and ipsilateral sides of the CA2 sub-region in cCCI animals to shams (*p* = 0.03; *p* = 0.02, respectively) (Figures 8B–D).

### Microglia morphology indicated regional heterogeneity in cCCI animals seven days following injury

**FIGURE 9:**
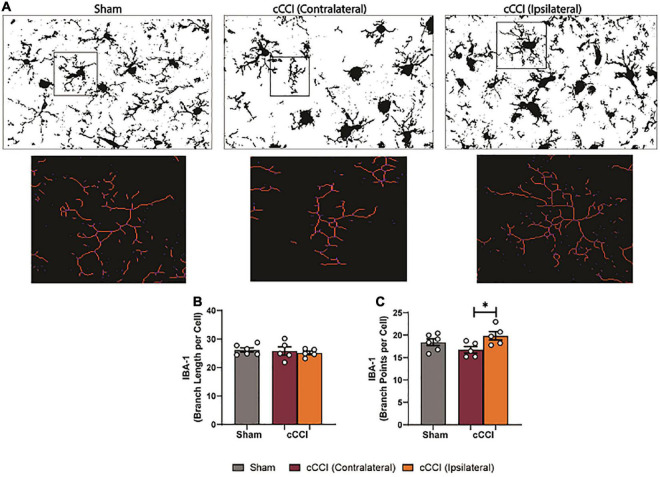
Increased microglial branching was present in the SC 7 days following TBI. **(A)** Representative images of microglia that were skeletonized for quantitative morphological analysis. **(B)** No significant changes were found between sham and cCCI animals for branch length per cell. **(C)** Significant increases in branch points per cell were observed in the ipsilateral SC compared to the contralateral SC of the cCCI group. *p < 0.05. Data represented as Mean ± SEM.

**FIGURE 10:**
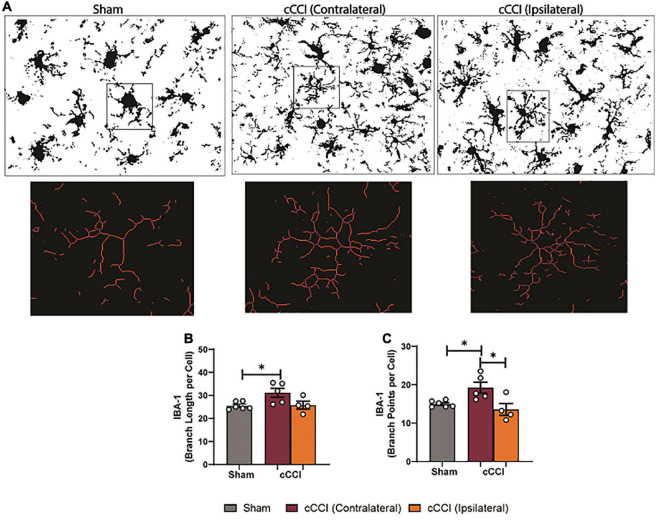
Changes in microglia processes were present following impact trauma. **(A)** Representative images show increases in branch length of microglia in the contralateral MC following injury, with increased branching within the ipsilateral MC. **(B)** Compared to shams, significant increases in branch length per cell were observed in the contralateral side of the MC. **(C)** Significant increases in branch points per cell were found in the contralateral MC compared to the ipsilateral side in the injured group, and were increased compared to the SC in the sham group. *p < 0.05. Data represented as Mean ± SEM.

Microglia morphology was quantified in the somatosensory and motor cortices and the hippocampal brain regions. Within the SC, a one-way ANOVA indicated significant differences in branch points per cell (*p* < 0.05), with an increase in branch points per cell observed in the ipsilateral side of the SC in the cCCI group compared to the contralateral side (*p* = 0.04). No significant changes were observed in the SC between groups when quantifying branch length per cell (Figures 9A–C). Within the MC, significant differences in branch length per cell were found with *post-hoc* tests showing a significant increase in branch length per cell in the contralateral side of the MC in the cCCI group compared to the MC of the sham group (*p* = 0.03). A significant increase in branch points per cell was also notable (Kruskal–Wallis test, *p* = 0.01), with Dunn’s multiple comparison test indicating this significant increase when comparing the contralateral side of the MC to the MC in the sham group (*p* = 0.04). A significant increase in branch points per cell was also observed in the contralateral side of the MC compared to the ipsilateral side within the injured group (*p* = 0.01) (Figures 10A–C).

While morphological changes in the hippocampus were found, these changes were limited as significance was only observed within the CA2 sub-region. A one-way ANOVA and *post-hoc* tests observed a significant increase in branch points per cell on the contralateral side of the CA2 compared to the CA2 of the sham group (*p* = 0.02). No significant changes were observed in any other sub-region for branch points per cell, and no significant differences were found within the hippocampus for branch length per cell.

### Decreased levels of NLRP3 were present within the cortex seven days following cCCI

**FIGURE 11:**
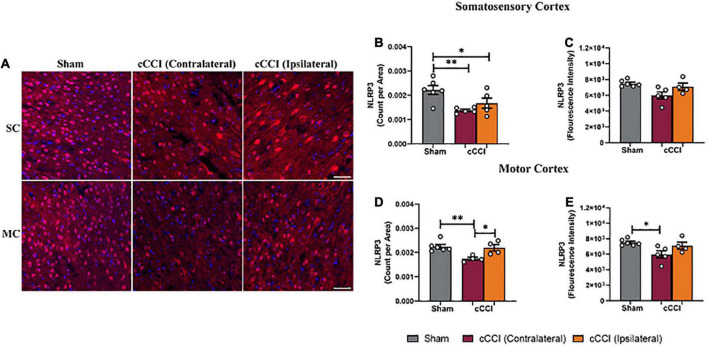
Decreased levels of NLRP3 were found in the cortex 7 days following impact. **(A)** Representative images of NLRP3 + molecules. Magnification = 20×. Scale bar = 100 μm. (Red) = NLRP3, (Blue) = DAPI. **(B)** Within the SC, decreased amounts of NLRP3 (count per area) were noted within both the contralateral and ipsilateral sides in the injured animals compared to the SC of shams. **(C)** No notable changes in fluorescence intensity were noted in the SC. **(D)** Count per area of NLRP3 in the ipsilateral MC was significantly increased compared to the contralateral side in the cCCI group, whereas count per area was significantly decreased in the contralateral MC of the injured animals compared to the MC of the sham group. **(E)** A significant decrease was noted in fluorescence intensity when comparing the contralateral side of the MC in the injured group to the shams. *p < 0.05 and ^**^p < 0.01. Data represented as Mean ± SEM.

Lower levels of NLRP3 were noticeable in the impact group compared to shams (Figure 11A). Furthermore, a one-way ANOVA indicated significant differences in the number of NLRP3 + molecules (count per area) in the SC (*p* < 0.05), with differences showing a significant decrease in count per area of NLRP3 in both the contralateral and ipsilateral sides of the SC in the injured group compared to the SC of the sham group (*p* = 0.003; *p* = 0.03, respectively). No significant difference in the fluorescence intensity of NLRP3 was observed within the SC (Figures 11B, C). In the MC, a one-way ANOVA indicated significant changes in NLRP3 production, with *post-hoc* multiple comparison tests showing a significant decrease in count per area in the contralateral side of the MC compared to the MC of the shams (*p* = 0.003). A significant decrease in count per area on the contralateral side of the MC in the cCCI group compared to the ipsilateral side was also observed (*p* = 0.01). Changes in NLRP3 expression (fluorescence intensity) were also found to be significant in the MC. More specifically, a significant decrease in the fluorescence intensity of NLRP3 was found within the contralateral side of the MC in the cCCI group compared to the MC of shams (*p* = 0.02) (Figures 11D, E). No significant changes were observed for any parameter for NLRP3 when analyzing the hippocampus region.

## Discussion

This study utilized a closed-head, closed-skull model of concussion in the adult rat using a CCI device (cCCI), was consistent with “mild” injury manifestations since it did not produce skull fracture yet resulted in significant delays in righting time. As a closed-head model, the cCCI offers critical advantages over existing preclinical TBI models. It can be used to understand the pathological changes following concussions, a common type of TBI that plagues many across the US and worldwide (Clay et al., 2013; Gardner and Yaffe, 2015; Kazl and Torres, 2019). Together, this is a valuable preclinical model emerging that is best suited to understand impact trauma and the repercussions accompanying this injury.

As concussion has been shown to elicit a myriad of symptoms within the acute and even chronic stages, continuing to understand the underlying mechanisms that drive these clinical symptoms is imperative. Astrogliosis and microglia activation are vital responses in secondary injury mechanisms following concussion, yet what drives the activation of these glial is still understudied. There are also very few closed-head impact models investigating glial contributions to pathology, with most of them exploring pathological changes following repeated concussion (Grant et al., 2018; Li et al., 2020; Vonder Haar et al., 2022). For example, astrocytes and microglia play a major role in the neuroinflammatory response following injury, with astrocytes and microglia releasing inflammatory cytokines. Microglia also depict unique morphological changes that aid in tissue plasticity, phagocytosis of cell debris, and interact with dysfunctional neurons (Linnerbauer et al., 2020; Mira et al., 2021).

In the current study, animals subjected to a cCCI were found to display glial-driven pathological responses 7 days following injury, with unique regional differences indicating glial responses that take place in not only the ipsilateral side of the brain but the contralateral side as well. The impact area was on the right side of the rat’s head, with the brain’s SC located underneath. Furthermore, the depth of impact and the width of the impact tip made it advantageous to explore the MC which is directly adjacent to the SC, and the hippocampus which is posterior to the cortex. Thus, these regions were further investigated when understanding glial response following the trauma. A notable response in GFAP was observed, with increased GFAP expression in the ipsilateral side of the SC compared to the contralateral side within the cCCI animals. While there was significance when comparing the side of impact (ipsilateral) and the opposite side of impact (contralateral), there were no notable changes in GFAP expression and the size of astrocytes in the cCCI animals when compared to their sham counterparts at 7 days. Furthermore, a decrease in GFAP + astrocytes was only found to be significant on the contralateral side of the SC in the cCCI animals compared to the SC of the sham animals. Studies focusing on understanding pathology following impact have noted changes usually within the ipsilateral side of the regions of interest. However, emerging research, as seen in the present study, shows changes within the contralateral side also ensuing (Grant et al., 2018; Yuan et al., 2020). A study by Yang et al. (2022) utilized CCI to understand regional impact trauma. They observed significant histological alterations of GFAP when comparing the ipsilateral side of the cortex and regions of the hippocampus compared to the sham group. However, changes were also observed when comparing the contralateral side to the shams. While astrocyte reactivity is traditionally identified as the proliferation of astrocytes in injured areas (Dimou and Götz, 2014; Li et al., 2019; Sofroniew, 2020), decreases in GFAP + astrocytes were observed at the impact site. Studies have reported on the loss of astrocytes and/or GFAP immunoreactivity following TBI, which may be due cell death, astrocytes proliferating to other injured areas to maintain homeostasis, or the breakdown of intermediate filaments and the accompanying alteration in overall protein function (Zhao et al., 2003; DeWalt et al., 2018; Frik et al., 2018; Zhou et al., 2020). Furthermore, a study by Shandra et al. (2019) noted an atypical astrocyte response following repetitive TBI where there was a subset of astrocytes that did not display classic signs of astrogliosis characterized by the upregulation of GFAP yet had rapid downregulation of homeostatic proteins and impaired astrocyte coupling. Diverse populations of astrocytes that do not display traditional signs of “reactivity” (upregulation of GFAP) could be present following cCCI, which could explain subacute decreases in GFAP expression and GFAP + astrocytes. Thus, future work will explore other astrocytic markers such as S100β, Kir4.1, and/or GLT1 to explore the theory that various subsets of astrocytes are present following cCCI, which may be contributing to the inflammatory response.

While the hippocampus was not the targeted site of impact, the contusive forces transduced through the impact caused significant changes, affecting GFAP expression and GFAP + astrocyte levels 7 days following impact. Throughout the sub-regions of the hippocampus, significant increases in GFAP expression and the size and number of astrocytes were notable on the ipsilateral side of the hippocampus compared to the contralateral side in the injured group. Additionally, astrocyte reactivity within the ipsilateral side was also apparent when compared to the hippocampus of the sham group. Reactivity of astrocytes has traditionally been seen as an increase in GFAP expression, proliferation of astrocytes, and hypertrophy of astrocytes (increased area per cell) which are all present factors within the impact side of the hippocampus following cCCI, with this response being markedly different from changes that were observed in the cortex. Astrocytes’ role in the hippocampus and the hippocampus’ sensitivity to injury and disease may explain why regional variances are observed. Astrocytes have a pivotal role in the neuro-repair process, primarily when regulating hippocampal synaptic plasticity (Ota et al., 2013; Lana et al., 2021; Wang et al., 2022). Astrocytes express multiple ion channels, neurotransmitter, and neuromodulator receptors, enabling them to sense the changes in neuronal activity around them. With impact, astrocytes actively respond to the changing environment around them, responding to the dysfunction of hippocampal neurons, which influences astrocyte reactivity. Furthermore, inflammatory responses from neurons and other glial cells within the hippocampus could be influencing reactivity seen within the ipsilateral side. A mouse model of open-head CCI indicated changes in astrocytes, with astrocyte reactivity evident in the ipsilateral hippocampus up to 2 weeks following injury (Semple et al., 2017), noting that increases in extracellular IL-1RA in the hippocampus released following CCI to be a leading contributor to inducing this reactivity. This specific inflammatory pathway within the hippocampus could be contributing to astrocyte changes that seem to be region-specific, with further studies in understanding cytokine release following closed-head CCI imperative.

A study by Prins et al. (2013) that characterized the cCCI model on juvenile rats indicated changes in astrocyte reactivity in the ipsilateral hippocampus, similar to the present study, with explanations indicating that vulnerability to cerebral hypometabolism, mitochondrial dysfunction, and oxidative injury may play a role in causing worsened astrocyte reactivity (Grant et al., 2018). However, the experimental design varied as the comparable study found these differences following a repeated mild TBI, which may influence vulnerability and signaling pathways that induce reactivity. In contrast, the present study indicated these differences within a single study. Additionally, the same model utilized by Jamnia et al. (2017) with parameters that utilized a 5 mm depth, angled impact, and a 10 mm depth, did not report on changes in astrocyte levels following impact, which indicates the importance of the continued studies within this CHI model, as changes in astrocyte reactivity seems to vary based on CCI parameters, which can make it challenging to determine injury severity.

A further indication of neuroinflammation following cCCI was also observed, with significant changes in AQP4 expression within the MC and the hippocampus. AQP4 plays a critical role in the brain, maintaining homeostasis through water balance, and is involved in astrocyte migration (Lu et al., 2013; Pittock, 2014; Ikeshima-Kataoka, 2016). Research has also indicated the direct role of AQP4 in neuroinflammation, with decreases in AQP4 indicating a reduction in inflammation following injury and disease (Fukuda and Badaut, 2012; Kitchen et al., 2020). AQP4 is most prevalent in astrocytic membranes, with increases in AQP4 expression indicative of astrocyte swelling, migration, edema, and BBB disruption. Within the present study, upregulation of AQP4 expression was present in the MC, with similar increases within the hippocampus’s CA1, CA2, and CA3 sub-regions in the ipsilateral and contralateral regions of interest within the cCCI group compared to shams. To our knowledge, no cCCI studies have explored AQP4 expression to understand its role in the pathological response following this injury and its contributions to neuroinflammation. Others have shown that increases in the protein levels of AQP4 following open-head CCI were a pivotal contributor to cerebral edema, a known sign of inflammation within the brain (Xiong et al., 2022). Additionally, a model of CCI for post-traumatic epilepsy also indicated that dysregulation of AQP4 leads to neurological deficits, which can be through the swelling of astrocytic endfeet (Szu et al., 2020; Lu et al., 2021). Theories have also found dysregulation of AQP4 to play a pro-inflammatory role contributing to neurological deficits in diseases such as Parkinson’s; thus, the role of AQP4 following closed-head impact TBI is important to study (Tamtaji et al., 2019; Prydz et al., 2020). While the experimental design of the compared studies may differ, the similarities in results, with both the present and a compared study showing increases in AQP4 following injury and disease, suggests that the closed-head aspect of this model still produces pathology that is representative of pathology reported in traditional CCI models that replicate pathology observed following mTBI in humans (Yao et al., 2015; Szu et al., 2020). The generated data is essential when establishing and optimizing closed-head injury models, and the continued utilization of cCCI will increase research into clinically relevant data.

The increases in AQP4 in the hippocampus may be associated with increased GFAP expression and increased size of astrocytes 7 days following impact. This data resembles research that has also identified that increases in AQP4 expression were related to astrocyte enlargement, which influences astrocyte reactivity (Ikeshima-Kataoka, 2016; Palazzo et al., 2019; Lu et al., 2021). As astrocyte reactivity plays a prominent role in the inflammatory response, neuronal dysfunction, and the metabolic changes that occur following injury, further exploration of the role of AQP4 and astrocytes, primarily through co-localization, is essential to understand this pathophysiology following cCCI in the future.

Seven days following impact, increases in IBA-1 expression were observed in the SC and MC of the cCCI animals compared to shams. Still, an overall decrease in the number of microglia cells was present within these regions. Results from Jamnia et al. (2017) observed changes in microglia distribution and morphology and found no significant differences 4 or 8 days following a single cCCI in the cortex, contrasting with the presence of microglia activation observed in the present study. Contrasting results could be due to variations in experimental design, such as differing injury parameters that may increase or decrease injury severity, or the fact that the contralateral and ipsilateral sides of the cortex were not delineated in the comparative study, which may dilute changes present in subacute microglia responses. Further, a study by Hubbard et al. (2018) indicated that changes in IBA-1 expression in injured animals compared to shams could indicate alterations in microglia phenotypes such as processes retraction. As these trends seem to be present in the current study, quantifying microglia morphology and changes in microglial phenotypes due to morphological changes was achieved. As quantified through skeleton analysis, significant changes in microglia morphology were found within the cortex 7 days following injury, which was found to be negligible in the compared study (Jamnia et al., 2017). In the SC, an increase in branch points per cell was observed within the ipsilateral side compared to the contralateral side, yet no significant differences were found for branch length per cell. Whereas in the MC, a substantial increase in branch length per cell and branch points per cell was found in the contralateral side of the MC compared to the MC of the sham group. With significant increases in area per cell, branch length per cell, and branch points per cell in the contralateral MC of the cCCI animals, microglia could be undergoing a dystrophic “bushy” morphology associated with microglia degeneration, with decreased surveillance and phagocytosis (anti-inflammatory).

Within the hippocampus, significant decreases in the number of microglia cells and IBA-1 expression were present in the DG, CA2, and CA3 sub-regions of the hippocampus. The hippocampus is quite susceptible to injury, with previous literature even demonstrating that the DG, CA2, and CA3 regions are particularly vulnerable to impact TBI because of increased neuronal loss, driving microglia activation (Hicks et al., 1993; Baldwin et al., 1997; Grady et al., 2003; Sandhir et al., 2008). Likewise, microglia depletion has also been linked to attenuating dendritic spine loss and neuronal apoptosis (Lier et al., 2021), which could explain why microglia depletion was observed in the DG, CA2, and CA3 of the present study. Moreover, other explanations for decreases in microglia and IBA-1 expression within the hippocampus could be attributed to apoptosis or microglia proliferating in other areas to maintain homeostatic function in response to impact. Future studies investigating neuronal dysfunction through dendritic and synapse loss that can drive microglia pathology are warranted to further explore hippocampal vulnerability in cCCI. Investigating specific markers such as Caspase-3 and TUNNEL and actin-related proteins such as coronins will also contribute to discovering whether microglia are dying in response to injury and insight into their subacute motility and migration patterns.

When quantifying morphological changes within the hippocampus, a significant decrease in the number of branch points was found within the contralateral side of the CA2 compared to the sham group. Still, no other changes were noted, such as an increase or decrease in cell body size or branch length per cell. A study by Hinwood et al. (2013) observed changes in microglia morphology following stress-induced pathology within the prefrontal cortex, revealing that stress increased the internal complexity of microglia. They saw enhanced ramification (increased branching) without altering the overall area occupied by the cell (cell body perimeter) or the length of the branches. Their results indicated that this specific morphology was not associated with increased pro-inflammatory cytokines. Instead, this phenotypic change was markedly different from those traditionally observed following injury. The unique morphology they observed was associated with an upregulation in β1-integrin (CD29), a protein involved in the ramification of microglia, most often upregulated in neurodegenerative diseases (Wu and Reddy, 2012; Hinwood et al., 2013). Similarities in morphology were notable in the CA2 sub-region in the cCCI group, meaning that microglia display a unique type of hyper-ramifciation in the hippocampus at the sub-acute stages, with a unique signaling pathway that may be driving this activation following cCCI.

Nucleotide-Binding Domain (NOD)-Like Receptor Protein 3 is an inflammasome that is primarily expressed on microglia. Levels of NLRP3 are a way to understand inflammation on a molecular level by acknowledging whether there is a dominant presence of pro or anti-inflammatory response within regions of interest. Within the SC and MC, the histological analysis indicated a significant decrease in NLRP3 protein levels in the contralateral and ipsilateral regions of the cCCI group compared to the cortices of the sham group. Morphological analysis of microglia indicated that dystrophic microglia, known to be anti-inflammatory, and losing their function, had a significant presence in the cortex 7 days following injury. If this is the dominant microglia phenotype within this area, this could explain why decreased levels of NLRP3 were observed in the cortex.

This study successfully provided insight into the sub-acute glial-induced inflammation in a closed-head model of CCI. Responses in the ipsilateral and contralateral sides of the regions of interest exhibited unique regional variances following impact trauma that needs to be studied further. In this case, the results revealed that while one side of the head is subjected to impact, dysfunction occurs in various brain regions regardless of the distance to the impact area. This could be due to impact loading subjecting forces onto the skull and brain that lead to a more diffuse injury, evidence that has been supported in mild impact injuries (Fehily and Fitzgerald, 2017). Moreover, the unique trans-hemispheric changes taking place such as reduction of astrocytes, increased expression of AQP4, and explicit changes in microglia morphology on the contralateral side of the brain could be due to anatomical and functional alterations that are developing in initially undamaged regions (Takatsuru et al., 2013; Young, 2014; Wiley et al., 2016). In a study by Le Prieult et al. (2017) following an open-head model of CCI, up to 4 days following injury, impairments in gamma-aminobutyric acid (GABA) ergic transmission and neuronal hyperactivity were observed in the contralateral somatosensory cortex, and glial reactivity in response to cellular debris were not restricted to the area of injury. The researchers of this study attributed neuronal activity in the contralateral side to an adapting mechanism to compensate for the functional loss of neuronal activity within the ipsilateral side of the brain. In the present study, changes in astrocyte reactivity and microglia morphology, as well as increases of AQP4 in the contralateral side of the brain could be linked to a transient response of glia interacting with neurons to compensate and stabilize the disrupted brain functions. While the experimental design of Le Prieult and colleagues’ study varied from ours, their results and theories may explain why glia alterations in distant regions of the brain are present within the current study. As the intrinsic and extrinsic properties of microglia, astrocytes, and neurons are interconnected to respond to injury, future studies that explore changes in neuronal dysfunction following cCCI and how glial functional states are altered as neural activity changes, can aid in supporting studies of the contralateral side of the brain compensating for disrupted brain functions following injury.

A limitation of this study was that, because the injury model was closed head, there is no explicit confirmation of the impact area. Most CCI models first expose the skull or go a step further and also use a craniotomy to identify the target site to assist in identifying a targeted site for the injury location. Unfortunately, in the closed head injury model, it is more challenging to locate bregma, which could introduce variability in the study’s outcomes. A skull template was used to mark the injury site in the specified coordinates. This template was a rat skull previously extracted from a rat of similar age and strain. A hole was drilled into the parietal bone (somatosensory cortex region of the brain), allowing for a mark on the top of the head of each animal. This approach allows for a close estimation of bregma each time. While this limitation is present, our studies have indicated consistent changes taking place in the regions of impact, adding confidence that the results are reproducible.

For the first time, this work has demonstrated the specific, unique roles that astrocytes and microglia display within a closed-head model of CCI within adult rats, with increasing evidence of specific biochemical responses and pathways that are mediated by glial cells in response to impact trauma. The results from this study are imperative in understanding the underlying mechanisms that contribute to adverse TBI outcomes and generating relevant data that can aid in improving diagnostic tools, rehabilitation, and therapeutic interventions for TBI patients.

## Data availability statement

The original contributions presented in this study are included in this article/supplementary material, further inquiries can be directed to the corresponding author.

## Ethics statement

This animal study was reviewed and approved by the Virginia Tech’s Institutional Animal Care and Use Committee.

## Author contributions

MW was responsible for study design, data collection, analysis of data, interpretation of results, and preparation of manuscript. PV was responsible for study design, securing funding, data collection, interpretation of results, and preparation of manuscript. Both authors contributed to the article and approved the submitted version.
